# Drinking water quality as a risk factor for pupils’ health in rural schools in Serbia

**DOI:** 10.1093/eurpub/ckac131.576

**Published:** 2022-10-25

**Authors:** D Jovanovic, V Karadzic, M Vasic, V Jovanovic, K Paunovic

**Affiliations:** 1 Institute of Public Health of Serbia, Belgrade, Serbia; 2 Faculty od Dentistry, Pancevo, Serbia; 3 Institute of Hygiene and Medical Ecology, Faculty of Medicine, University of Belgrade, Belgrade, Serbia

## Abstract

**Background:**

Access to safe drinking water in schools is essential for a good health, wellbeing, learning and dignity of pupils. The rural schools are most frequently connected to the rural water supply system or own water supply source, where the monitoring of drinking water quality is scarced. The study was conducted in the rural regions in Serbia in 2016, aiming at assessing drinking water quality and sanitary conditions of school water supply facilities.

**Methods:**

In total 238 school facilities, including 108 in Šumadija and 130 in Pomoravlje District were investigated using laboratory testing of drinking water and sanitary inspection.

**Results:**

Study revealed that 32% of analysed samples showed microbiological faecal contamination with bacteria E. coli, while 52% of samples showed physico-chemical non-compliance with the national standards, where higher nitrates were the most common cause. Overall non-compliance of drinking water from school rural water supply systems amounted to 66% of tested water samples. This study also showed the main technical shortcomings of school water supply facilities and distribution networks such as the lack of fencing of the water source, damaged or absence of drainage channels from the well concrete floor and poor construction characteristics of wells.

**Conclusions:**

The results of this study, particularly presence of E. coli in drinking water may affect pupils’ health and is a consequence of poor sanitary conditions and maintenance of small rural drinking water supply systems and individual wells. It is necessary to provide continuous disinfection of water available in schools and to undertake measures for technical improvements in order to provide better sanitary protection of water sources and other water supply facilities in rural schools.

**Key messages:**

• Drinking water quality in schools is vital for children public health.

• Raise awareness of local community on drinking water safety and enforce continuous monitoring of drinking water quality in small rural water supply systems in schools.

